# Towards Water Soluble Mitochondria-Targeting Theranostic Osmium(II) Triazole-Based Complexes

**DOI:** 10.3390/molecules21101382

**Published:** 2016-10-18

**Authors:** Salem A. E. Omar, Paul A. Scattergood, Luke K. McKenzie, Helen E. Bryant, Julia A. Weinstein, Paul I. P. Elliott

**Affiliations:** 1 Department of Chemistry, University of Huddersfield, Queensgate, Huddersfield HD1 3DH, UK; Salem.Omar@hud.ac.uk (S.A.E.O.); P.Scattergood@hud.ac.uk (P.A.S.); 2 Department of Chemistry, Dainton Building, University of Sheffield, Sheffield S3 7HF, UK; lkmckenzie1@sheffield.ac.uk (L.K.M.); julia.weinstein@sheffield.ac.uk (J.A.W.); 3 Academic Unit of Molecular Oncology, Sheffield Institute for Nucleic Acids (SInFoNiA), Department of Oncology and Metabolism, University of Sheffield, Beech Hill Road, Sheffield S10 2RX, UK; h.bryant@sheffield.ac.uk

**Keywords:** triazole, osmium, photophysics, complexes, ligands, anticancer, oxygen sensitizer

## Abstract

The complex [Os(btzpy)_2_][PF_6_]_2_ (**1**, btzpy = 2,6-bis(1-phenyl-1,2,3-triazol-4-yl)pyridine) has been prepared and characterised. Complex **1** exhibits phosphorescence (λ_em_ = 595 nm, τ = 937 ns, φ_em_ = 9.3% in degassed acetonitrile) in contrast to its known ruthenium(II) analogue, which is non-emissive at room temperature. The complex undergoes significant oxygen-dependent quenching of emission with a 43-fold reduction in luminescence intensity between degassed and aerated acetonitrile solutions, indicating its potential to act as a singlet oxygen sensitiser. Complex **1** underwent counterion metathesis to yield [Os(btzpy)_2_]Cl_2_ (**1^Cl^**), which shows near identical optical absorption and emission spectra to those of **1**. Direct measurement of the yield of singlet oxygen sensitised by **1^Cl^** was carried out (φ (^1^O_2_) = 57%) for air equilibrated acetonitrile solutions. On the basis of these photophysical properties, preliminary cellular uptake and luminescence microscopy imaging studies were conducted. Complex **1^Cl^** readily entered the cancer cell lines HeLa and U2OS with mitochondrial staining seen and intense emission allowing for imaging at concentrations as low as 1 μM. Long-term toxicity results indicate low toxicity in HeLa cells with LD50 >100 μM. Osmium(II) complexes based on **1** therefore present an excellent platform for the development of novel theranostic agents for anticancer activity.

## 1. Introduction

Oligopyridyl complexes of kinetically inert d^6^ metals, e.g., Ru(II), Os(II), have attracted enormous interest in recent decades due to their attractive photophysical properties [1,2,3,4]. These complexes typically exhibit relatively long-lived triplet metal-to-ligand charge transfer (^3^MLCT) states. These states may undergo deactivation through a number of routes including phosphorescence or energy/electron transfer which enables the potential application of these complexes in light-emitting [5] and photovoltaic technologies [6]. Key to the development of complexes for these applications is the design of the ligands supporting these metals. We, and others, have paid particular attention to the use of copper-catalysed coupling of alkynes and azides to form 1,2,3-triazole-based ligands [7,8,9,10] and have investigated the photophysical properties of their resultant complexes. A significant number of reports have appeared detailing the photophysical and photochemical properties of triazole-based complexes of Re(I), Ru(II) and Ir(III) [11,12,13,14,15,16,17,18,19,20,21,22,23,24,25]. Examples of triazole-containing complexes of osmium(II) are, however, comparatively rare. We have recently reported the synthesis and characterization of deep-red/near-IR emissive osmium(II) bitriazolyl (btz) complexes and demonstrated their use in light-emitting electrochemical cells [19].

Of recent and growing interest has been the use of luminescent complexes in biological applications [26,27,28]. Since phosphorescence is longer-lived than the autofluorescence from biological organic compounds, emissive complexes are amenable to use in time-gated imaging microscopy [29,30,31]. Osmium(II) complexes, however, often exhibit lower energy spin-forbidden direct ^3^MLCT optical absorption bands of moderate extinction coefficient due to the high spin-orbit coupling constant for the osmium centre [32,33]. This offers the advantage of enabling efficient excitation at lower energies that therefore avoids potential cellular damage and negates autofluorescence and the necessity of the added expense of time-gated apparatus. These absorption and emission bands at wavelengths closer to the red in comparison to those of common iridium(III) complexes, and therefore in a more biologically transparent region of the spectrum, will also enable greater depth of penetration for excitation and imaging. Complexes of osmium(II) are also typically highly inert to ligand photosubstitution making them highly robust [34] (although the unprecedentedly facile ligand photoejection in the complex [Os(btz)_3_]^2+^ was recently reported [35]). The intensity of phosphorescence is, however, sensitive to the presence of oxygen resulting in quenching through conversion of ground state ^3^O_2_ to reactive ^1^O_2_, thus enabling exploitation in photodynamic therapy (PDT) [36]. These combined properties thus present significant opportunities for the development of unique dual-mode theranostic agents.

We report here the synthesis and characterization of the orange-emissive bis(terdentate) osmium(II) complex [Os(btzpy)_2_]^2+^ (btzpy = 2,6-bis(1-phenyl-1,2,3-triazol-4-yl)pyridine) as its hexafluorophosphate (**1**) and chloride (**1^Cl^**) salts. The complex shows significant dependence of emission intensity on the presence of oxygen. The water soluble chloride salt **1^Cl^** has been subjected to preliminary cellular uptake and luminescence imaging studies and relevant results are reported.

## 2. Results & Discussion

**Scheme 1:**
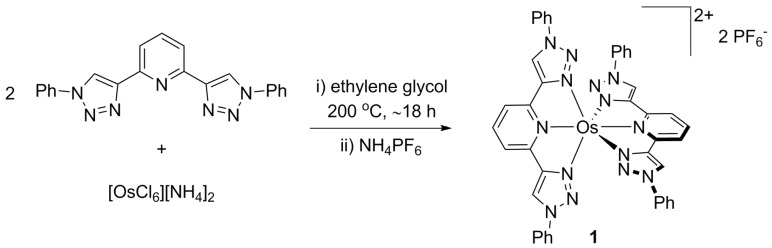
Synthesis of [Os(btzpy)_2_][PF_6_]_2_ (**1**).

Complex **1** was prepared by reaction of two equivalents of the ligand btzpy [37,38] with [OsCl_6_][NH_4_]_2_ in refluxing ethylene glycol (Scheme 1). After being left to cool, the complex was isolated as an orange powder, its hexafluorophosphate salt, through treatment with NH_4_PF_6_. The ^1^H-NMR spectrum of **1** exhibits a characteristic singlet resonance for four equivalent triazole ring protons at δ 9.13, which is deshielded relative to the corresponding signal for the free ligand by 0.16 ppm. The protons of the central pyridine ring give rise to doublet and triplet resonances at δ 8.36 and 8.01, respectively, with those for the phenyl substituents resulting in multiplets between δ 7.50 and 7.60. For spectroscopic comparison the complex [Os(tolterpy)_2_][PF_6_]_2_ (**2**, tolterpy = 4′-*p*-tolyl-2,2′:6′,2′′-terpyridine) was also prepared.

The electrochemical properties of **1** were investigated by cyclic voltammetry (CV) and reveal a reversible Os(II)/Os(III) oxidation at +0.64 V (vs Fc/Fc^+^ = 0.0 V). This is close to that exhibited by the known model complex **2** (E^ox^ = +0.49 V) and other related osmium(II) complexes [39,40,41] indicating that the highest occupied molecular orbital (HOMO) has primarily metallic 5d orbital character. Unlike in the CV trace for **2**, no ligand-based reductions are observed for **1** within the available electrochemical window (−2.0 to +1.2 V), which is indicative of the higher energy lowest unoccupied molecular orbital (LUMO) localized on the btzpy ligand compared to that for the tolterpy complex. This LUMO destabilisation is consistent with previously reported data on the ruthenium(II) analogue of **1** versus [Ru(terpy)_2_]^2+^ (terpy = 2,2′:6′,2′′-terpyridine) [42,43,44].

**Figure 1:**
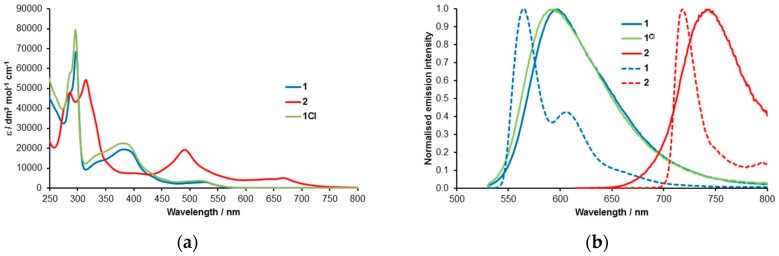
(**a**) UV-visible absorption spectra for **1** and **2** in acetonitrile solutions and **1^Cl^** in aqueous solution at room temperature; (**b**) normalized emission spectra of complexes **1** and **2** in de-aerated acetonitrile solutions and **1^Cl^** in aerated aqueous solution at room temperature (solid lines) and complexes **1** and **2** in 4:1 EtOH/MeOH glasses at 77 K (dashed lines).

UV-visible absorption spectra were recorded for **1** and **2** in acetonitrile solutions at room temperature (Figure 1a and Table 1). The spectrum of **1** exhibits a strong absorption at 297 nm assigned to ligand-centred π→π* transitions localized on the btzpy ligand along with a broad band between 350 and 400 nm assigned to ^1^MLCT transitions. This is significantly blue-shifted relative to that observed for **2** (491 nm) consistent with the btzpy ligand, and hence its complex, having a much higher energy LUMO as indicated from the CV data described above. Similarly to the data for **2**, complex **1** also exhibits absorptions of lesser intensity at longer wavelengths corresponding to spin-forbidden direct ^3^MLCT excitations (λ^max^ = 526 nm) from the singlet ground state enabled by the large spin-orbit coupling constant associated with the osmium centre [32,33].

In stark contrast to its ruthenium(II) analogue, **1** is emissive at room temperature in de-aerated acetonitrile solutions, with the emission being characterized by a broad featureless band at 595 nm (λ^ex^ = 500 nm) and a lifetime of 937 ns (Figure 1b and Table 1) attributed to an emissive ^3^MLCT state. Bis(tridentate) complexes of ruthenium(II) typically show little or no emission at room temperature as the deviation from an ideal octahedral-like coordination geometry results in stabilization of triplet metal-centred (^3^MC) states relative to the ^3^MLCT state [46,47]. As such, ^3^MC states are efficiently populated from photoexcited ^3^MLCT states thereby quenching emission. The observed emission for **1** must therefore arise from the destabilization of the ^3^MC states due to the typically larger ligand-field splitting associated with the 5d metal centre over its 4d counterpart, such that non-radiative depopulation of the emissive ^3^MLCT state is comparatively disfavoured. Mirroring the UV-visible absorption data, the emission spectra of **1** are significantly blue-shifted relative to those of **2** by 143 nm (3260 cm^−1^) indicative of the comparatively destabilized LUMO for **1**, and hence also its ^3^MLCT state. The emission spectrum was also recorded at 77 K in a 4:1 EtOH/MeOH glass matrix and shows a structured emission band that is blue-shifted relative to the spectrum at room temperature due to rigidochromic effects.

**Figure 2:**
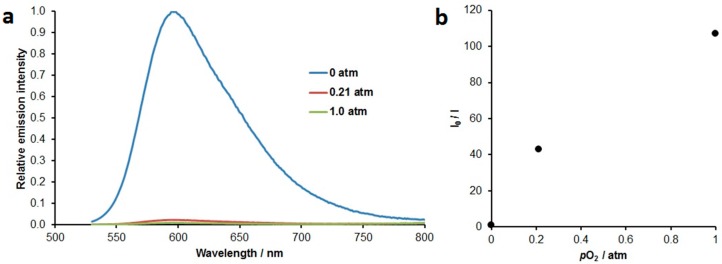
(**a**) Relative oxygen dependent emission spectra for **1** in acetonitrile (normalized for deoxygenated (vacuum) conditions) and (**b**) Stern-Volmer plot (*I*_0_ and *I* are the emission intensities at λ_max_ for the degassed solution and solution at the partial pressure of oxygen at which emission is measured respectively).

Emission intensity from **1** is dramatically affected by the presence of oxygen (Figure 2) and is quenched by approximately 43-fold when recorded in air compared to deaerated conditions. The long lifetime of emission combined with the oxygen sensitivity confirms the assignment of a ^3^MLCT-based emissive state. This significant quenching of emission by oxygen thus presents the possibility of utilizing complexes based on **1** as potential ^1^O_2_ sensitizers for photodynamic therapeutic applications.

**Figure 3:**
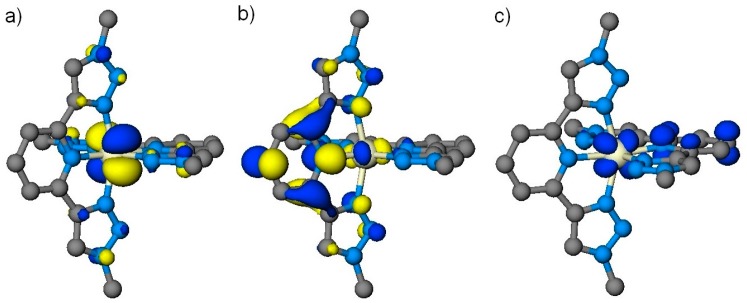
Plots of the HOMO (**a**) and LUMO (**b**) for the ground state of **1** and the spin density for the T_1_ state of **1** (**c**).

Complex **1** was also studied by density functional theory (DFT) calculations to confirm the nature, localisation and relative energies of the frontier orbitals as well as to simulate the optical absorption spectrum. The data reveal that the HOMO is localized primarily on the osmium(II) centre as expected (Figure 3a) but with a small contribution from the π-systems of the four triazole rings. The LUMO is localized on one of the btzpy ligands, predominantly on the central pyridine ring and with a lesser contribution from the triazole rings (Figure 3b) but also a metallic d-orbital contribution. The HOMO of **1** is slightly stabilized (−10.63 eV) relative to that of **2** (−10.35 eV) in agreement with the experimental electrochemical data. The LUMO (−6.95 eV) on the other hand is significantly destabilized relative to that of **2** (−7.32 eV) due to the smaller π-system associated with the btzpy ligand compare to tolterpy and due to the electron rich triazole moieties. This results in a larger HOMO–LUMO gap for **1** of 3.68 eV compared to that for **2** (3.03 eV) mirroring the significantly blue-shifted absorption and emission data.

Time-dependent DFT was used to calculate the lowest energy 30 singlet state vertical excitations at the ground state geometry along with the lowest energy 10 spin-forbidden triplet excitations for **1**. The data agree well with the experimental spectra (Supporting Information) but with a slight overestimation of the energies of transitions compared to bands in the UV-visible absorption spectrum. The S_1_ state is calculated to have an energy of 2.74 eV (452 nm) and is primarily HOMO → LUMO ^1^MLCT in character. The first major transition (S_7_, 374 nm) is predominantly composed of a HOMO **→** LUMO+2 transition and is similarly of ^1^MLCT character confirming our experimental assignment of the band in this region of the UV-visible absorption spectrum. The T_1_ transition is calculated to be at 512 nm (2.42 eV), is of mixed HOMO-2 **→** LUMO+1 and HOMO-1 → LUMO character and is therefore in agreement with the assignment of the lesser intensity absorptions between 450 and 550 nm as arising from spin-forbidden direct ^3^MLCT transitions.

The lowest lying triplet state of **1** was optimized starting from the optimized ground state geometry and is calculated to lie 2.40 eV above the energy of the ground state. The spin density was plotted and is presented in Figure 3c. It reveals unpaired electron density on both the metal and one of the btzpy ligands confirming the ^3^MLCT character of this T_1_ state. Curiously, unlike in the case of **2**, the T_1_ state of **1** undergoes a puckering like distortion of the btzpy ligand on which the unpaired electron density is localized. Such distortions have been observed, however, in theoretical calculations of the T_1_ states of bis(tridentate) ruthenium(II) cyclometalated complexes [48] and [Os(terpy)_2_]^2+^ [49].

Conversion to the chloride salt, [Os(btzpy)_2_]Cl_2_ (**1^Cl^**), was achieved by stirring a suspension of **1** in methanol with Amberlite IRA-400 ion-exchange resin (chloride form) before filtering, removal of solvent and freeze-drying from aqueous solution. Removal of the hexafluorophosphate couterion was confirmed by the lack of the corresponding resonances in the ^19^F- and ^31^P-NMR spectra. The UV-visible absorption spectrum of **1^Cl^** (Figure 1) in aqueous solution is near identical to that of its analogous hexafluorophosphate salt **1** in acetonitrile. The complex is also emissive in aerated aqueous solution with an emission maximum at 589 nm, very slightly blue-shifted relative to that of **1** in acetonitrile.

**Figure 4:**
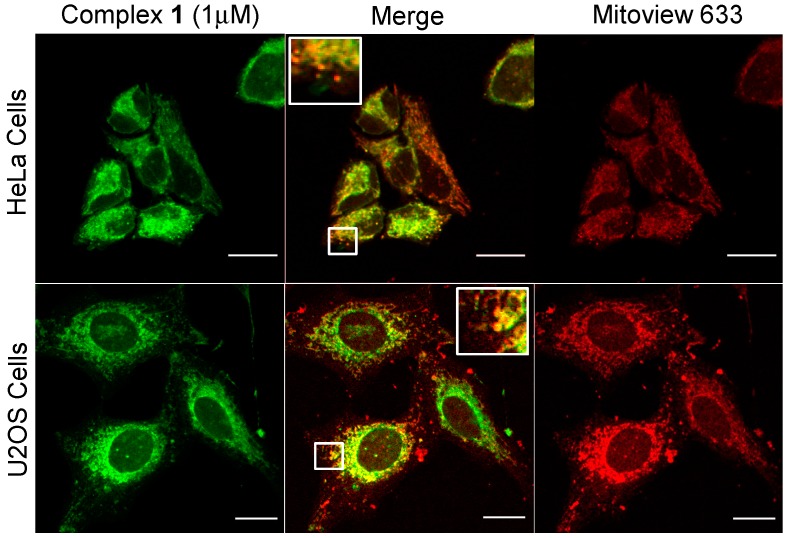
Confocal images of complex **1^Cl^** (green) following 4 h incubation in HeLa and U2OS cells co-localised with Mitoview 633 (red) with central overlaid image, scale bars 20 μm.

Based on the highly encouraging photophysical data reported above we decided to carry out preliminary studies on cell uptake and toxicity. Complex **1^Cl^** was seen to localise to the mitochondria in the cancer cell lines HeLa (cervical cancer) and U2OS (osteosarcoma) following a short incubation time of 4 h and with clear phosphorescence seen at concentrations as low as 1 μM. Colocalisation with the mitochondrial stain MitoView 633 was seen under confocal microscopy (Figure 4), giving Pearson′s correlation coefficients of r = 0.85 and 0.7 for HeLa and U2OS cells respectively. A Pearson′s correlation coefficient of 1 indicates complete concurrence of the stains, while 0 indicates no concurrence hence these values indicate that complex **1^Cl^** preferentially localises to the mitochondria.

**Figure 5:**
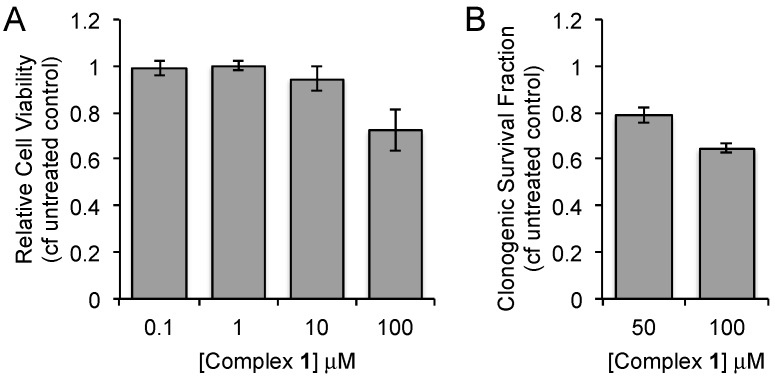
(**A**) MTT and (**B**) Clonogenic survival assays following incubation of HeLa cells with increasing concentrations of complex **1^Cl^** in the dark. In each case, mean and standard deviation of at least 3 independent repeats is shown.

The cellular viability of HeLa cells following incubation with complex **1^Cl^** at concentrations up to 100 μM was assessed by MTT assay (Figure 5). In addition, long-term survival was assessed using clonogenic assays and indicated an LD_50_ > 100 μM in dark conditions. This shows that at a concentration effective for luminescence imaging microscopy the complex is non-cytotoxic, lending support to potential use as a PDT agent where non-toxicity in the dark is desired.

The yield of singlet oxygen generation, φ (^1^O_2_), of complex **1^Cl^** was measured in air-equilibrated acetonitrile against the standard perinaphthenone. A φ (^1^O_2_) of 57% was determined by direct measurement of the ^1^Δ_g_ state emission from ^1^O_2_ in the NIR (λ_em_ 1275 nm) under 355 nm irradiation by a pulsed Nd:YAG laser as described previously [50]. It is proposed that apoptotic cell death after light treatment is associated with localisation of photosensitizers to mitochondria [51]. Thus the sub-cellular localisation, long-term survival following treatment of cancer cells with complex **1^Cl^** in the dark and the high singlet oxygen yield of 57% indicate the potential for this complex as a photosensitizer for PDT theranostic applications. We recognize that the wavelengths of absorption for **1^Cl^** are at relatively high energy compared to the ideal for a PDT agent and thus are not at the optimum position for maximum tissue penetration for excitation. However, modification of the basic design of the complex by making the ligand more electron-withdrawing, for example, should be a relatively easy task. Stabilisation of the LUMO, thus decreasing the HOMO-LUMO gap, would shift the electronic absorption of the complexes into the desired lower energy region of the spectrum. Based on these encouraging results, work is currently underway to fully determine the anticancer activity of **1^Cl^** and for the further development of **1^Cl^** and analogues thereof as a new class of potential PDT agents.

## 3. Conclusions

We have reported the synthesis, characterisation and photophysical properties of a novel luminescent osmium(II) triazole-based complex. The complex has been shown to exhibit significant quenching of luminescence intensity in the presence of oxygen and a high quantum yield for singlet oxygen sensitization as its chloride salt, indicating potential applications in photodynamic therapy as well as luminescence imaging microscopy. The water soluble chloride form of the complex was subjected to preliminary cellular uptake and luminescence imaging microscopy studies. The results from these studies reveal that the complex is successfully taken up by two cancer cell lines with mitochondrial localization and low dark toxicity.

The use of CuAAC coupling in ligand synthesis opens up diverse avenues for tailored derivitisation and bioconjugation that would enable the optimisation of cellular uptake and a wider scope for organelle targeting within cells. Combined with the attractive photophysical properties, the complex described in this contribution represents a highly versatile platform for the development of dual-mode luminescence imaging/singlet oxygen sensitisation photodynamic theranostic complexes. Plans to pursue these studies are in progress and results from these studies will be published elsewhere in due course.

## 4. Experimental Section

### 4.1. General Methods

Ammonium hexachloroosmate(IV) was purchased from Alfa Aesar (Ward Hill, MA, USA) whilst all other reagents were purchased from Sigma-Aldrich (Saint Louis, MO, USA), Acros Organics (Thermo Fisher Scientific, Geel, Belgium) or Fluorochem (Hadfield, UK) and used as supplied. The ligand btzpy [37] and complex **2** [52] were prepared by literature procedures. NMR spectra were recorded on a Bruker Ascend 400 MHz spectrometer (Billerica, MA, USA), with all chemical shifts being quoted in ppm referenced relative to the residual solvent signal (MeCN, ^1^H: δ = 1.94, ^13^C: 1.32, 118.26; DMSO, ^1^H: δ = 2.50, ^13^C: 39.52 ). High-resolution mass spectrometry was performed on an Agilent 6210 TOF instrument (Santa Clara, CA, USA) with a dual ESI source. UV-visible absorption spectra were recorded on an Agilent Cary 60 spectrophotometer whilst emission spectra were recorded on a Fluoromax 4 spectrophotometer (aerated and degassed in acetonitrile and data at 77 K in a 4:1 EtOH/MeOH glass). Lifetime measurements were performed using an Edinburgh instruments Mini-Tau spectrometer (Edinburgh, UK). Emission quantum yields (φ_em_) were measured for degassed MeCN solutions, with degassing carried out via three repeat freeze-pump-thaw cycles. Quantum yields are quoted relative to [Ru(bpy)_3_][PF_6_]_2_ in aerated MeCN, with analyte solutions being excited at a single wavelength at a point of common optical density. Thus, φ_em_ values are determined from the ratio of integrated area under the corrected peaks, with an assumed experimental uncertainty of ±20%. Cyclic voltammograms were recorded using an Autolab PGSTAT100N potentiostat with NOVA electrochemical software (version 1.10.1.9). Analyte solutions were prepared using nitrogen saturated dry acetonitrile, freshly distilled from CaH_2_. All measurements were conducted at room temperature under a stream of dry nitrogen at potential scan rates ranging from 20 to 500 mV·s^−1^. [NBu_4_][PF_6_] was used as a supporting electrolyte, being recrystallised from ethanol and oven dried prior to use, with a typical solution concentration of 0.2 mol dm^−3^. The working electrode was a platinum disc, with platinum wire utilised as the counter electrode. The reference electrode was Ag/AgCl, being chemically isolated from the analyte solution by an electrolyte containing bridge tube tipped with a porous frit. Ferrocene was employed as an internal reference, with all potentials quoted relative to the Fc^+^/Fc couple

### 4.2. Synthesis of [Os(btzpy)_2_][PF_6_]_2_ *(**1**)*

Ammonium hexachloroosmate(IV) ([(NH_4_)_2_OsCl_6_], 150 mg, 0.341 mmol) and 2.5 equivalents of 2,6-bis(1-phenyl-1*H*-1,2,3-triazol-4-yl)pyridine (310 mg, 0.85 mmol) in ethylene glycol (25 cm^3^) was heated at reflux overnight under nitrogen. The resulting mixture was then allowed to cool to room temperature. 2.5 equivalents of aqueous NH_4_PF_6_ was added resulting in a dark brown precipitate which was collected by filtration and washed with cold water and diethyl ether. This was then redissolved in acetonitrile, cooled in the fridge overnight and filtered to remove unreacted ligand. The solvent was removed from the filtrate and the residue recrystallized from dichloromethane/hexane to give an orange powder. Yield = 185 mg, 45%; ^1^H-NMR (400 MHz, CD_3_CN): δ 9.13 (s, 4H); 8.36 (d, *J* = 8.0 Hz, 4H); 8.01 (t, *J* = 8.0 Hz, 2H); 7.49–7.60 (m, 20H). ^13^C-NMR (101 MHz, CD_3_CN): δ 153.26, 151.74, 138.26, 136.94, 131.12, 131.07, 124.98, 121.64, 120.18. ESI HRMS: calculated for [C_42_H_30_N_14_Os]^2+^ *m*/*z* = 461.1191; found *m*/*z* = 461.1195.

### 4.3. Synthesis of [Os(btzpy)_2_]Cl_2_ *(**1^Cl^**)*

A suspension of **1** (100 mg, 0.083 mmol) in methanol (25 cm^3^) was stirred with Amberlite IRA-400 ion-exchange resin (chloride form, 200 mg) for 24 h at R.T. in the dark. The resin was removed by filtration and the solvent then removed by evaporation. The residue was then dissolved in water and the solution was freeze dried to yield **1^Cl^** as an orange powder. Yield = 71 mg, 87 % ^1^H-NMR (400 MHz, *d*_6_-DMSO): δ 10.15 (s, 4H); 8.58 (d, *J* = 8.0 Hz, 4H); 8.21 (t, *J* = 8.0 Hz, 2H); 7.66–7.72 (m, 8H); 7.47–7.57 (m, 12H). ^13^C-NMR (101 MHz, *d*_6_-DMSO): δ 152.01, 150.32, 137.57, 135.58, 130.08, 130.00, 125.27, 120.46, 119.31. ESI HRMS: calculated for [C_42_H_30_N_14_Os]^2+^ *m*/*z* = 461.1191; found *m*/*z* = 461.1192.

### 4.4. Computational Details

The geometries of cations for complexes **1** and **2** were optimized using DFT calculations at the B3LYP [53,54] level of theory (20% Hartree-Fock). Phenyl substituents of the btzpy ligand were simplified to methyl groups to reduce computational cost. The Stuttgart-Dresden relativistic small core effective pseuopotential and basis set was used for osmium [55] and 6-311G* basis sets used for all other atoms [56]. Optimised minima were confirmed through vibrational frequency calculations. TDDFT calculations were carried out at the optimized ground state geometries to compute the vertical excitation energies (lowest 30 singlet and 10 triplet roots) and hence the simulated optical absorption spectra. The T_1_ states were also optimized and the spin density calculated and plotted. All calculations were carried out using the NWChem 6.6 software package [57] with geometries, molecular orbital surfaces and spin densities viewed and plotted using the ECCE graphical user interface.

### 4.5. Cell Culture

Both HeLa (human cervical cancer) and U2OS (human bone osteosarcoma) cell lines were purchased from American Type Culture Collection–LGC partnership (Teddington, UK) and used within 20 passages of purchase. Cells were cultured in Dulbecco′s modified Eagles Medium (DMEM) (Lonza, Cambridge, UK) with 10% fetal calf serum (FCS) (Lonza, Cambridge, UK) and incubated at 37 °C under 5% CO_2_. Both cell lines were routinely checked for mycoplasma infection. Complex **1^Cl^** was stored as a stock solution at 10 mM in DMSO.

### 4.6. Luminescence Imaging and Colocalisation Studies

Cover glasses (22 × 22 mm) were sterilised (industrial methylated spirits, IMS) and placed flat in 6-well plates. Cells were seeded at a density of ~1 × 10^5^ cells per well and allowed to adhere overnight in culture media. Complex **1^Cl^** (1 μM) was added and incubated for 4 h, for co-localisation studies MitoView™ 633 (Biotium) was added for the final 15 min, prior to cells being washed 3 times in PBS and fixed in 4% paraformaldehyde solution in PBS at 4 °C for 20 min. Following a further wash (PBS × 3) the coverslips were mounted to microscope slides (IMMU-MOUNT™, Life Technologies Ltd, Paisley, UK). The slides were imaged by confocal microscopy (Nikon A1 confocal) using a 60× lens (CFI Plan Apochromat VC 60× oil, NA 1.4). An argon laser (405 nm and 561 nm) was used to excite complex **1^Cl^** and a diode laser (642 nm) was used to excite MitoView™ 633. Colocalisation indices were calculated using the open source imaging software Fiji (based on ImageJ) and the coloc 2 colocalisation tool. The threshold regression chosen was Bisection.

### 4.7. Cell Viability Assay-MTT

96-well plates were seeded with HeLa cells at 1000/well and incubated overnight. Wells were treated with 0.1–100 μM complex **1^Cl^** or DMSO control and incubated for 4 h before replacing with fresh media. After 5 days further growth, 25 μL of 3 mg cm^−3^ thiazoyl blue (MTT) solution was added to each well. Following incubation for 3 h the solution was removed from each well and 250 μL/well DMSO added ensuring mixing of crystals. Optical density of wells at 540 nm was recorded on a plate reader (Multiskan fc, Thermo Fisher Scientific, Warrington, UK).

### 4.8. Clonogenic Survival

Six-well plates were seeded with HeLa cells at 400 cells/well and incubated overnight. Wells were treated with DMSO, 50 μM or 100 μM complex **1^Cl^** for 4 h before replacing with fresh media. Plates were incubated for 8–10 days to form colonies before staining with 4% methylene blue in 70% methanol and counting. Each colony was considered to represent a single surviving cell and survival fraction calculated for each condition compared to DMSO control.

## Figures and Tables

**Table 1:** Summarised photophysical data for **1**, **1^Cl^** and **2** in acetonitrile.

Complex	λ^abs^/nm ^1^ (ε/dm^3^·mol^−1^·cm^−1^)	λ^em^/nm ^1^	τ/ns ^2,3^	φ_em_/% ^2,4^	λ^em^/nm ^5^
**1**	526 (3025), 434 (5700), 382 (19,500), 337 (13,500), 297 (68,500), 288 (49,000)	595 ^6^	937 ± 12	9.3	564, 606 ^6^
**1^Cl^**	534 (3315), 438 (5800), 390 (24,750), 345 (17,350), 297 (90,800), 287 (62,500)	599 ^6^ (589) ^6,8^	884 ± 6 (273 ± 3) ^8^	9.7 (5.4) ^8^	-
**2**	669 (5070), 645 (4600), 491 (1930), 406 (7520), 314 (54,300), 286 (48,000)	738 ^7^	339 ± 4	3.2	718, 795 ^7^

^1^ RT, acetonitrile solutions; ^2^ Degassed MeCN at RT; ^3^ λ^ex^ = 405 nm; ^4^ Relative to [Ru(bpy)_3_][PF_6_]_2_ φ_em_ = 0.018 in aerated MeCN [45]; ^5^ 77 K, 4:1 EtOH/MeOH glass; ^6^ λ^ex^ = 500 nm; ^7^ λ^ex^ = 600 nm; ^8^ Aerated aqueous solution.

## Supplementary Materials

The following are available online at www.mdpi.com/1420-3049/21/10/1382/s1, Figure S1: ^1^H-NMR spectrum of **1** (CD_3_CN), Figure S2: ^13^C-NMR spectrum of **1** (CD_3_CN), Figure S3: ESI mass spectrum of **1**, Figure S4: ^1^H-NMR spectrum of **1^Cl^** (*d*_6_-DMSO), Figure S5: ^13^C-NMR spectrum of **1^Cl^** (*d*_6_-DMSO), Figure S6: ESI mass spectrum of **1^Cl^**, XYZ coordinates for the optimized geometries of the ground and lowest lying triplet states of **1**, Figure S7: Time-dependent DFT UV-visible absorption spectrum of **1**, Table S1: Summarised time-dependent DFT data for **1**.
