# Supplementary material for: Towards Water Soluble Mitochondria-Targeting Theranostic Osmium(II) Triazole-Based Complexes
Source: Molecules. 2016 Oct 18;21(10):1382. doi: 10.3390/molecules21101382 (PMC6273092; doi:10.3390/molecules21101382)
Supplement: Supplementary file 1 [file molecules-21-01382-s001.pdf]

Supplementary Materials: Towards Water Soluble Mitochondria-Targeting Theranostic Osmium(II) Triazole-Based Complexes

Salem A. E. Omar, Paul A. Scattergood, Luke K. McKenzie, Helen E. Bryant, Julia A. Weinstein and Paul I. P. Elliott

Figure S1	^{1}H-NMR spectrum of $[Os(btzpy)_2][PF_6]_2$ (**1**)	**S2**
Figure S2	^{13}C-NMR spectrum of $[Os(btzpy)_2][PF_6]_2$ (**1**)	**S3**
Figure S3	ESI mass spectrum of $[Os(btzpy)_2][PF_6]_2$ (**1**)	**S3**
Figure S4	^{1}H-NMR spectrum of $[Os(btzpy)_2][Cl]_2$ ($\mathbf{1}^{Cl}$)	**S4**
Figure S5	^{13}C-NMR spectrum of $[Os(btzpy)_2][Cl]_2$ ($\mathbf{1}^{Cl}$)	**S4**
Figure S6	ESI mass spectrum of $[Os(btzpy)_2][Cl]_2$ ($\mathbf{1}^{Cl}$)	**S5**
DFT optimised ground and triplet state geometries for complex $[Os(btzpy)_2]^{2+}$		**S5–7**
Figure S7	TDDFT spectrum for $[Os(btzpy)_2]^{2+}$	**S7**
Table S1	Summary of TDDFT vertical excitation data for $[Os(btzpy)_2]^{2+}$	**S8**

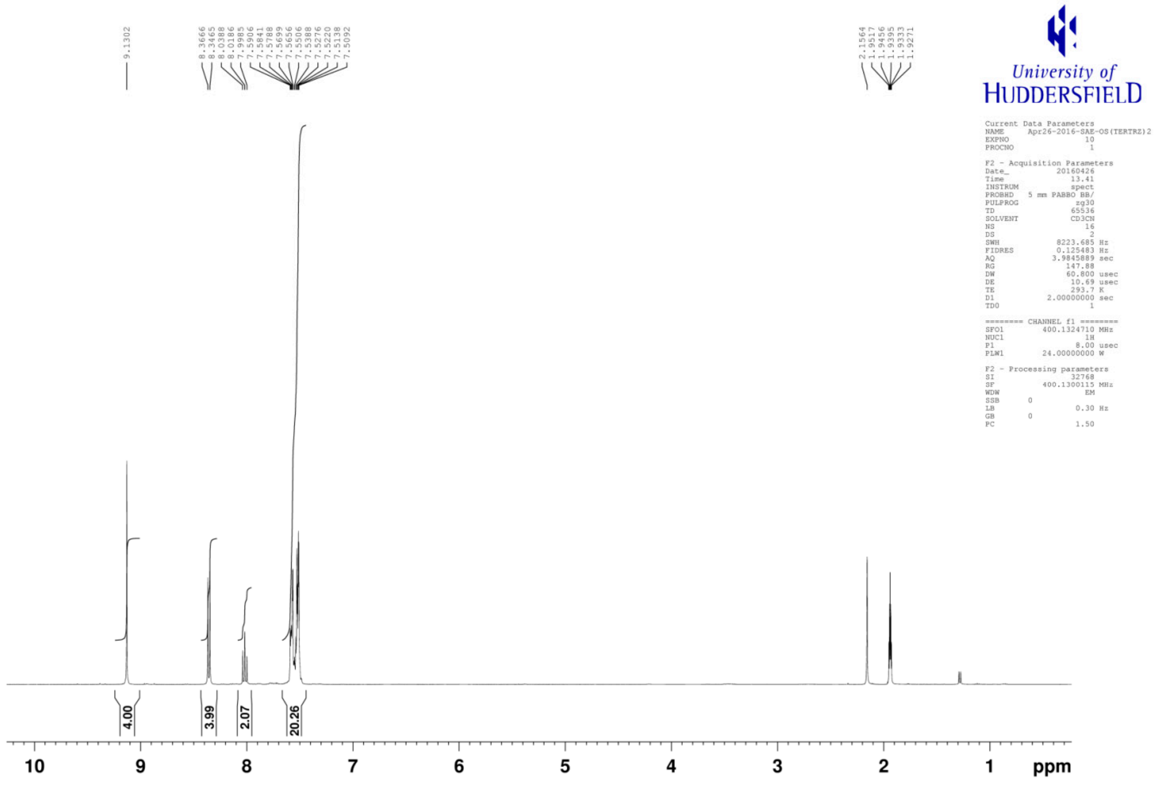

Figure S1. ^{1}H-NMR (400 MHz, d_3-MeCN) spectrum of **1.**

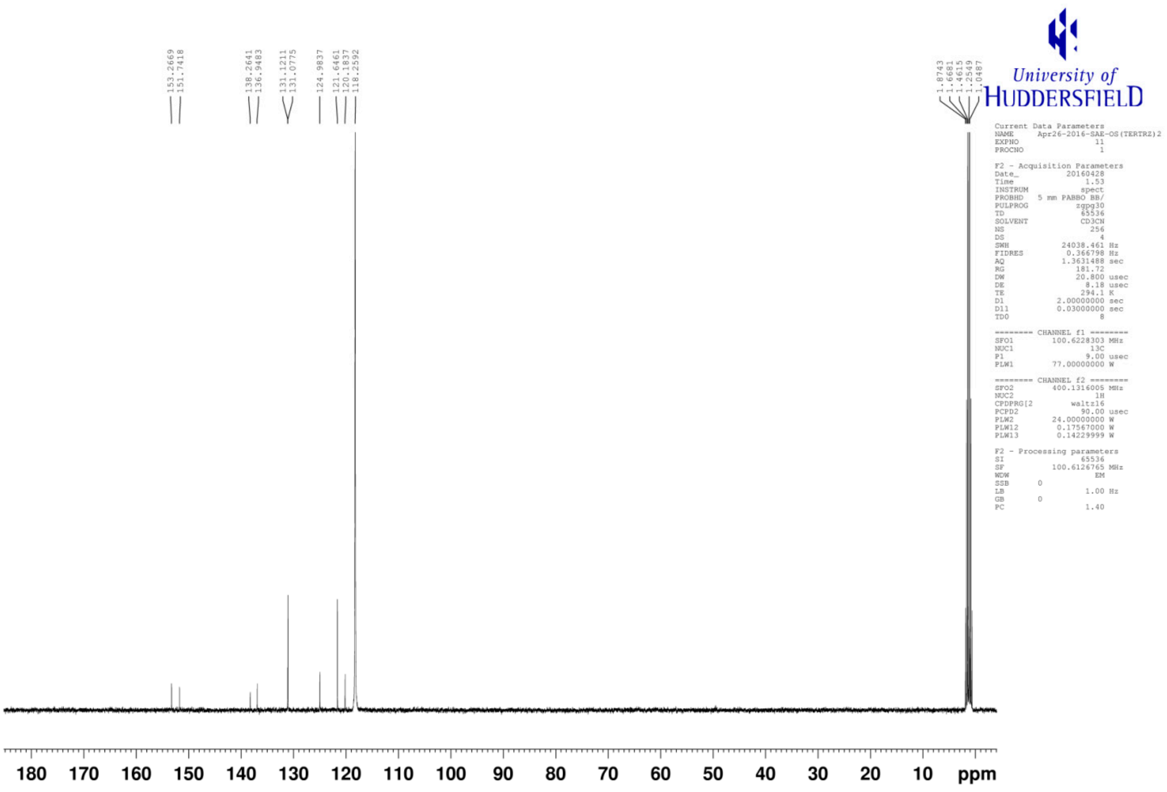

Figure S2. ^{13}C-NMR (101 MHz, d_3-MeCN) spectrum of **1**.

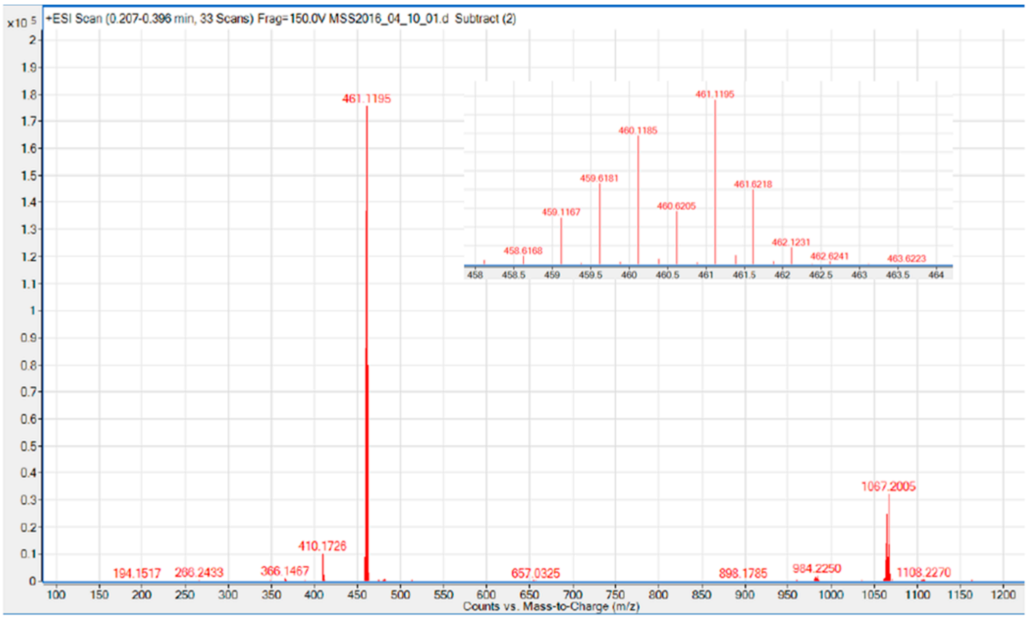

Figure S3. High resolution ESI mass spectrum for **1**. The expansion shows detail of the $[C_{42}H_{30}N_{14}Os]^{2+}$ mass fragment. The cationic ion pair $\{[C_{42}H_{30}N_{14}Os][PF_6]\}^{+}$ is also observed at m/z = 1067.2005.

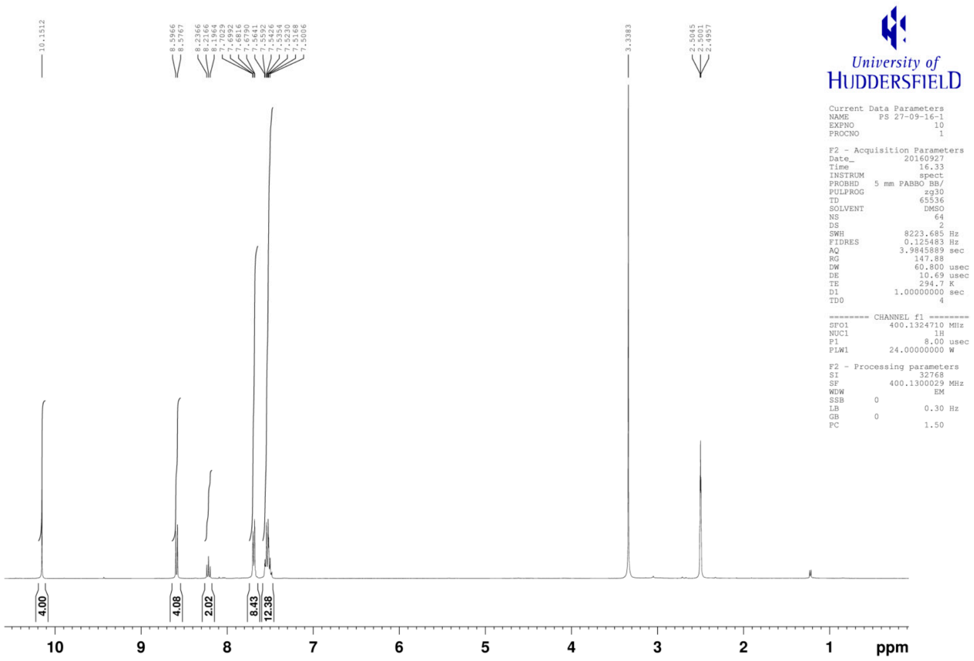

Figure S4. ^{1}H-NMR (400 MHz, d_6-DMSO) spectrum of **1^{Cl}.**

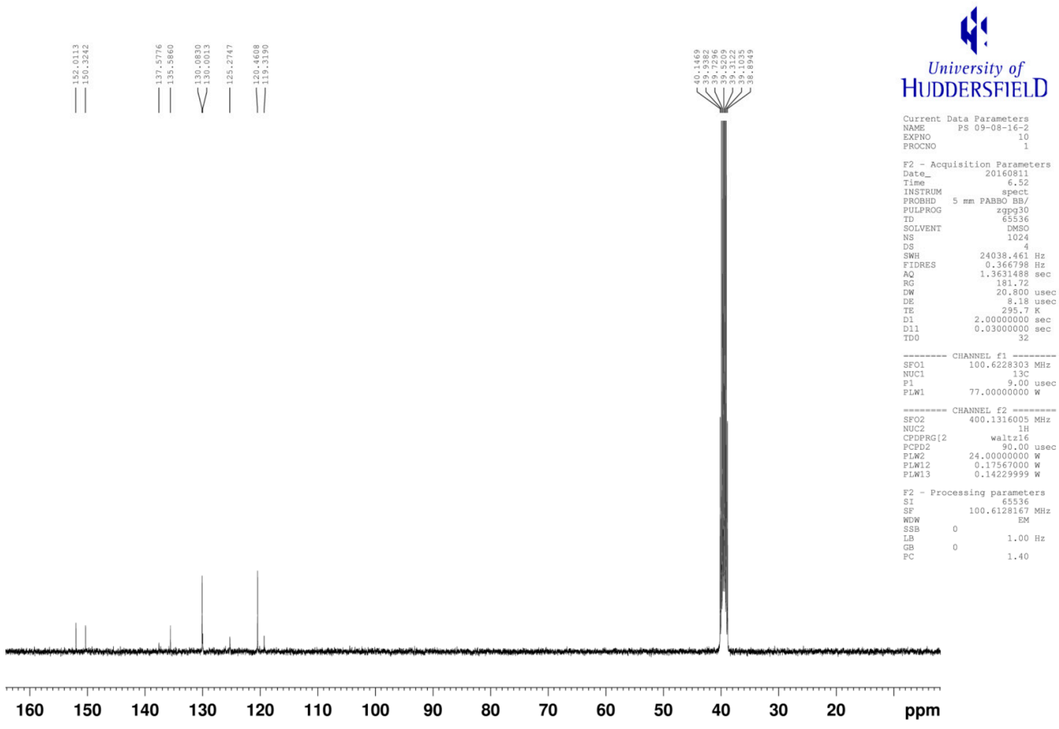

Figure S5. ^{13}C-NMR (101 MHz, d_6-DMSO) spectrum of **1**^{Cl}.

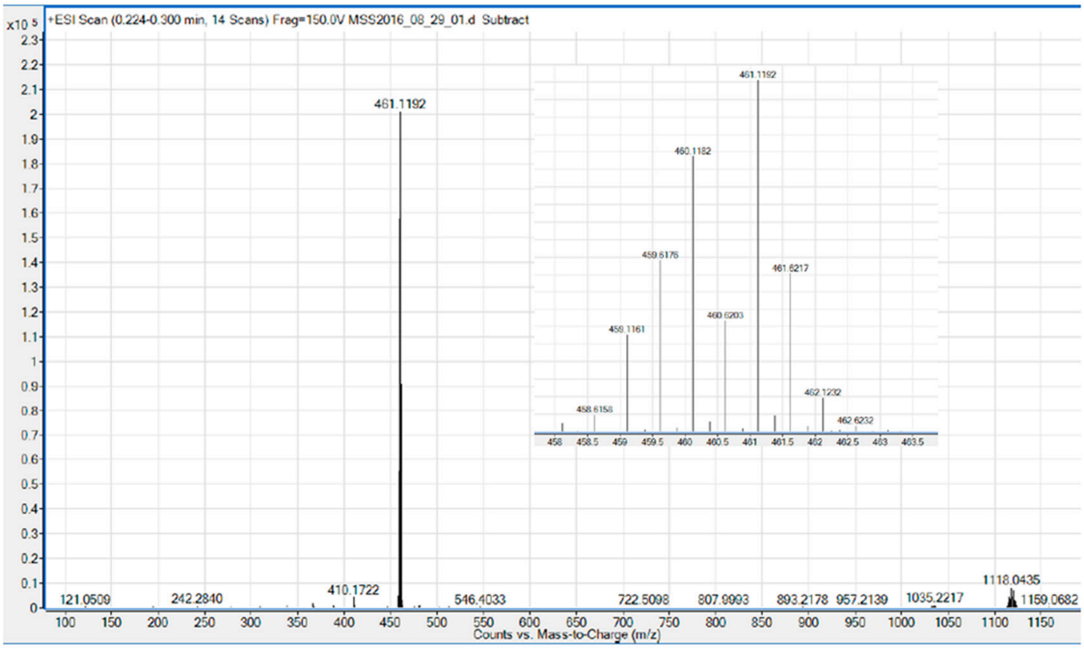

Figure S6. High resolution ESI mass spectrum for **1**^{Cl}. The expansion shows detail of the $[C_{42}H_{30}N_{14}Os]^{2+}$ mass fragment.

XYZ coordinates for optimised ground state geometry of $[Os(btzpy)_2]^{2+}$ 59 geometry

	X	Y	Z
Os	0.00037831	−0.00001671	−0.00008369
N	1.48264883	−0.12295165	1.46999247
N	2.29736703	0.72406015	2.03942651
N	3.01804563	0.01669161	2.91790722
C	2.66892556	−1.2899966	2.91845714
C	1.66756779	−1.39534131	1.97744192
C	0.83097035	−2.46139716	1.44131815
N	−0.02359735	−1.99876293	0.49370528
C	−0.89294883	−2.8312213	−0.13359452
C	−1.71035973	−2.12010421	−1.1087362
C	−2.71344175	−2.44521485	−1.99516912
N	−3.03515725	−1.28169018	−2.60671588
N	−2.29531113	−0.26179738	−2.15431557
N	−1.49580768	−0.76177555	−1.25178148
C	0.83438869	−3.80830032	1.79238141
C	−0.05396724	−4.67170946	1.15434565
C	−0.92723986	−4.18602293	0.1828913
C	4.02704546	0.69206609	3.73684752
C	−4.04369914	−1.04996694	−3.64359979
N	1.40189648	0.06726901	−1.55078507
N	2.1491102	−0.81016009	−2.16499462
N	2.8448869	−0.13045263	−3.08442634
C	2.54785027	1.18891361	−3.06603382
C	1.60696958	1.33210683	−2.06924251
C	0.84342458	2.4290595	−1.48830074
N	0.02594091	1.99869016	−0.49392353
C	−0.77423249	2.86336333	0.18009887
C	−1.56260154	2.18323509	1.20020895
C	−2.50222493	2.54560248	2.1401122
N	−2.83440401	1.39456286	2.76942337
N	−2.1599621	0.34759072	2.27830621
N	−1.39285411	0.81750203	1.33253247

	X	Y	Z
C	0.87924764	3.77524191	−1.84032893
C	0.06191062	4.67156629	−1.1545342
C	−0.77384617	4.2188361	−0.13534276
C	3.78516365	−0.84230102	−3.95277932
C	−3.78729148	1.20164495	3.86520168
H	3.14114267	−2.01380642	3.56208934
H	−3.20468181	−3.37537268	−2.23020295
H	1.51582873	−4.17892157	2.54848559
H	−0.06585776	−5.72345311	1.41424004
H	−1.6205536	−4.85138372	−0.31712287
H	4.5550336	−0.05648754	4.32314467
H	3.54009097	1.40462658	4.40112195
H	−4.02379283	0.00872691	−3.88756102
H	−5.02918396	−1.32372633	−3.26805246
H	3.01028169	1.89454266	−3.73640209
H	−2.94372618	3.49366728	2.40087242
H	1.53121642	4.12016332	−2.63366341
H	0.07615963	5.72328718	−1.41440255
H	−1.41193161	4.90987606	0.40197644
H	3.24543538	−1.58145116	−4.5426841
H	4.26088583	−0.12076665	−4.61299764
H	−3.8289726	0.13689908	4.07811661
H	−4.77218191	1.5565091	3.56360409
H	4.72818758	1.21165081	3.08625597
H	−3.80474771	−1.63746502	−4.52959181
H	4.53930127	−1.33513133	−3.34143677
H	−3.44972652	1.74196887	4.7492682

XYZ coordinates for the optimised lowest lying triplet excited state of $[Os(btzpy)_2]^{2+}$ 59 geometry

	X	Y	Z
Os	0.01845474	0.05781669	0.00311501
N	1.487627	−0.13390125	1.47527065
N	2.30251278	0.7193301	2.03057555
N	3.02865984	0.01763692	2.90468257
C	2.68040783	−1.28961336	2.91656835
C	1.67215299	−1.40155498	1.983473
C	0.83114242	−2.46335998	1.446889
N	−0.02039133	−1.99850151	0.50270458
C	−0.89027523	−2.82323231	−0.12465495
C	−1.70627285	−2.11167488	−1.10055267
C	−2.71220975	−2.42962844	−1.98703209
N	−3.0263115	−1.26188092	−2.59537426
N	−2.28126894	−0.24978654	−2.14299045
N	−1.4878702	−0.75955324	−1.24429782
C	0.83193939	−3.81007075	1.79844818
C	−0.05984851	−4.66867977	1.15920366
C	−0.93108701	−4.17901941	0.18856806
C	4.02308557	0.70537757	3.73505074
C	−4.02978959	−1.01971962	−3.63779239
N	1.41894882	0.08266841	−1.56374972
N	2.18263713	−0.81356737	−2.14639259
N	2.86850636	−0.15426764	−3.07729122
C	2.55698427	1.16405887	−3.10825899
C	1.60650924	1.33812164	−2.1177948
C	0.83631262	2.42666423	−1.56528026
N	−0.0589103	1.96641059	−0.57158615

	X	Y	Z
C	−0.84669776	2.88026464	0.16755446
C	−1.60737091	2.20561649	1.19160787
C	−2.55424763	2.54094395	2.14287712
N	−2.84150185	1.38214696	2.7854459
N	−2.14628318	0.35372511	2.30527282
N	−1.40038345	0.84358919	1.34240081
C	0.88743925	3.763787	−1.86305922
C	0.05949259	4.67412968	−1.1650922
C	−0.79230857	4.2170422	−0.1332031
C	3.79949331	−0.88186826	−3.94247632
C	−3.78255495	1.17628266	3.88845911
H	3.1576729	−2.0095593	3.56091616
H	−3.20933686	−3.35632013	−2.2233939
H	1.51252787	−4.18319503	2.55386396
H	−0.07568169	−5.72081876	1.41771876
H	−1.62678045	−4.84051918	−0.31293626
H	4.73097551	−0.02972337	4.11150259
H	3.52596791	1.20460159	4.56628225
H	−4.03615131	0.04747465	−3.84203218
H	−5.00991856	−1.33496388	−3.28222831
H	3.01483209	1.84808	−3.80306851
H	−3.02719024	3.47405889	2.40056053
H	1.56562274	4.12857224	−2.62598337
H	0.09807315	5.72877853	−1.40382789
H	−1.39408277	4.92708645	0.42251583
H	3.24273948	−1.48723282	−4.65707507
H	4.41676614	−0.16036098	−4.4728357
H	−3.77765799	0.11724956	4.13150312
H	−4.78238445	1.48047899	3.58042375
H	4.5417356	1.43634042	3.11924542
H	−3.76241636	−1.56836439	−4.54030406
H	4.42860114	−1.52107094	−3.3266109
H	−3.46763749	1.75644661	4.75544338

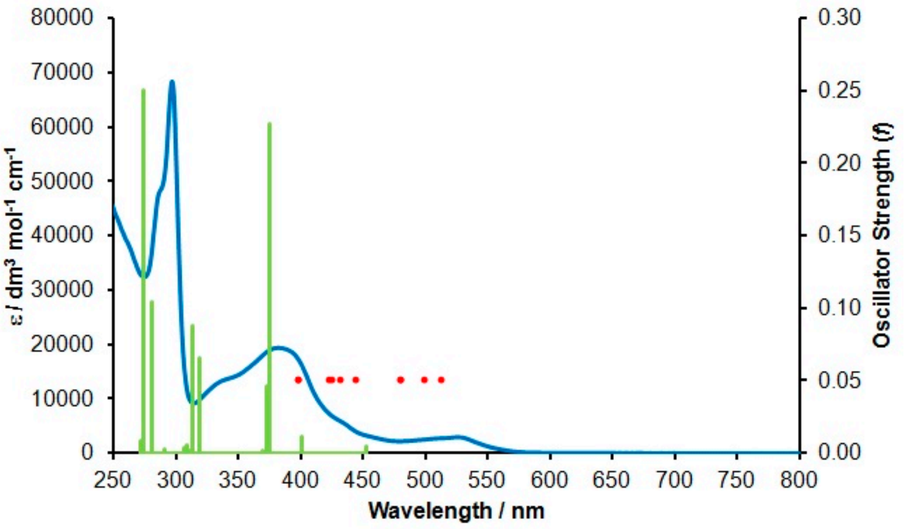

Figure S7. Simulated UV-visible absorption spectrum of $[Os(btzpy)_2]^{2+}$: singlet vertical excitations from TDDFT calculations (green trace) with experimental spectrum overlaid (blue trace). Red dots indicate positions of calculated spin-forbidden direct triplet excitations.

Table S1. Summarised data for major singlet excitations and the three lowest energy triplet excitations from TDDFT calculations.

State	λ/nm (Energy/eV)	Oscillator Strength (*f*)	Composition	Character
S_1	452 (2.74)	0.004	HOMO → LUMO (96%)	[1]MLCT
S_7	374 (3.31)	0.227	HOMO → LUMO+2 (34%)<br>HOMO-2 → LUMO+1 (13%)<br>HOMO-1 → LUMO (13%)	[1]MLCT
S_{13}	319 (3.36)	0.065	HOMO → LUMO+4 (88%)	[1]MLCT
S_{14}	313 (3.89)	0.088	HOMO → LUMO+5 (88%)	[1]MLCT
S_{27}	274 (4.53)	0.251	HOMO-4 → LUMO (63%)<br>HOMO-4 → LUMO+1 (15%)	[1]LC
T_1	512 (2.42)	-	HOMO-2 → LUMO+1 (44%)<br>HOMO-1 → LUMO (44%)	[3]MLCT
T_2	499 (2.48)	-	HOMO-2 → LUMO+1 (25%)<br>HOMO-1 → LUMO (25%)<br>HOMO-2 → LUMO (21%)<br>HOMO-1 → LUMO+1 (21%)	[3]MLCT
T_3	480 (2.58)	-	HOMO → LUMO (76%)<br>HOMO → LUMO+1 (20%)	[3]MLCT
